# Cost-utility analysis of centrally inserted totally implanted access port (PORT) vs. peripherally inserted central catheter (PICC) in the oncology chemotherapy

**DOI:** 10.3389/fpubh.2022.942175

**Published:** 2022-07-22

**Authors:** Guoliang Shao, Xiaoying Zhou, Shaoya Zhang, Shuaijun Wu, Yichen Dong, Zuojun Dong

**Affiliations:** ^1^Department of interventional oncology, The Cancer Hospital of the University of Chinese Academy of Sciences (Zhejiang Cancer Hospital), Institute of Basic Medicine and Cancer, Chinese Academy of Sciences, Hangzhou, China; ^2^Institute of Pharmaceutical Preparations, Zhejiang University of Technology, Huzhou, China; ^3^Faculty of Chinese Medicine, Macau University of Science and Technology, Macau, China

**Keywords:** central venous catheter, peripherally inserted central catheter (PICC), centrally inserted totally implanted access port (PORT), quality of life, cost-utility analysis, chemotherapy

## Abstract

**Background:**

Peripherally inserted central catheter (PICC) and centrally inserted totally implanted access port (PORT) are two types of intravenous infusion devices that are widely used in clinical practice. PORTs are more expensive to insert than PICCs but have fewer complications. Two cost-utility analyses of PICCs and PORTs in China have been published, but had conflicting findings. This study aimed to compare the cost-utility of PICCs and PORTs.

**Methods:**

We conducted a prospective observational trial including 404 patients with cancer and a cross-sectional study to calculate cost and complications of a PICC and PORT. Utility was measured using the EuroQol five-dimensional questionnaire (EQ-5D-5L). A cost-utility analysis was performed from a healthcare system perspective in China.

**Results:**

The average total cost of PICCs and PORTs were ¥ 4,091.7 and ¥ 4,566.8, which yielded 0.46 and 0.475 quality-adjusted life-years (QALYs) in a 6-month dwell time, respectively. The incremental cost-utility ratio (ICUR) was ¥ 31,670.9 per QALY. A one-way sensitivity analysis showed that the base-case results were robust, and the probabilistic sensitivity analysis showed that at a willingness-to-pay (WTP) threshold of ¥ 80,976 per QALY (China's per capita GDP in 2021) the probability of a PORT being cost-effective was 96%.

**Conclusion:**

PORTs were more cost-effective than PICCs for a 6 and 12-month dwell time. The total cost for a PORT was also less than that of a PICC. PORT is therefore recommended as a medium to long-term intravenous delivery device in clinical practice.

## Introduction

The rate of cancer diagnosis continues to rise in China. In 2017, the total cancer expenditure for Chinese residents reached RMB 304.84 billion, with a per capita treatment cost of RMB 50,000 (1). Chemotherapy is currently one of the most effective methods for treating cancer. However, the repeated venous punctures needed for chemotherapy may lead to vascular injury and most chemotherapy drugs have strong irritant and corrosive effects if extravasated, resulting in side effects such as phlebitis (2). To protect the patient's blood vessels from corrosive chemotherapeutic drugs and reduce their pain, central venous catheters are widely used in clinical practice (3). In addition to delivering chemotherapeutic drugs, central catheters can also be used for bolus or maintenance nutrient solutions, drugs or blood products (4).

Centrally inserted totally implanted access ports (PORT) and peripherally inserted central catheters (PICC) are two widely used medium- and long-term intravenous infusion devices. Both can safely infuse stimulating drugs while protecting the patient's blood vessels (5–7). Many clinical studies have shown that the probability of PICC-related complications is higher than that of PORT-related complications (8–10), in particular with respect to retention time and the increased pain of repeated venous punctures (11). However, since the cost of PORT implantation is twice that of PICC, PICCs are used more often clinically. Comparative cost analyses of these two catheterization techniques have been performed (12–14), but the health outcomes of patients who receive these two types of catheters have not. There are currently two studies in China that have performed a comparative cost-utility analysis for these catheters (15, 16). However, they reported opposite findings, and the cost of PORT insertion has decreased as the centralized purchase catalog continues to be adjusted. The pharmacoeconomic evidence regarding these two techniques must be updated to ensure appropriate clinical and health care decision-making.

This study prospectively collected the complication rates, direct medical costs and health outcomes associated with PICC and PORT used in individual patients. We then calculated the incremental cost-utility ratio (ICUR) of these two placement methods from the perspective of the healthcare system and at a threshold of China's GDP per capita in 2021 in order to measure the economic impact of these catheters.

## Materials and Methods

### Study design and participants

Patients with PICCs and PORTs implanted at a tertiary-referral hospital in Zhejiang from April 6, 2021 to May 6, 2021 were selected for this study. Inclusion criteria were: (1) patients ≥18 years old; (2) oncology patients requiring long-term intravenous infusion; (3) initial PICC or PORT placement; and (4) no contraindications to the implantation of PICC or PORT. Exclusion criteria were: (1) clinically significant upper extremity/central deep venous thrombosis; (2) unable to communicate or suffering from psychiatric disease. This study lasted for 1 year. Demographic and clinical information, costs, health outcomes and patient data such as age, gender and disease diagnosis were collected prospectively from the electronic case system. Complication rates were calculated based on follow-up data. A cost-utility analysis was performed using health economics methods, with the primary endpoint being the removal of the catheter. The PORT and PICC groups were enrolled according to clinical practice, with no alterations in patient care throughout the study. For study purposes, the PICC group was considered the control group and the PORT group was the experimental group.

Ethical approval was granted by the Cancer Hospital of The University of the Chinese Academy of Sciences (IRB-2020-11). Informed consent was obtained from all patients participating in the study.

### Adverse effects

Adverse effects were collected starting the day after catheter placement. The main complications associated with central venous catheterization are shown in Table 1. Patients were followed up 1, 3, 6, and 12 months after PICC or PORT insertion.

**Table 1:** Adverse effects.

**Adverse effects**	**PICC (*****n*** **=202)**	**PORT (*****n*** **=202)**
Catheter-related thrombosis	5 (2.5%)	1 (0.5%)
Catheter occlusion	12 (5.9%)	8 (4%)
Migration	12 (5.9%)	1 (0.5%)
Infection	5 (2.5%)	5 (2.5%)
Eczema	21 (10.4%)	4 (2%)
Other	4 (2%)	6 (3%)

*Data are presented as n (%). PICC, peripherally inserted central catheter; PORT, Centrally inserted totally implanted access port*.

### Cost

Only direct medical costs were considered from the perspective of the Chinese healthcare system. Cost information was collected in four parts: insertion cost, maintenance cost, complication cost and removal cost. All costs were measured by the Cancer Hospital of The University of Chinese Academy of Sciences. Insertion and removal costs were one-time costs. As the maintenance cycle is different for PICCs and PORTs, with PICCs being maintained once a week and PORTs once a month, the maintenance cost was equal to single maintenance cost × maintenance times. The common management measures for catheter-related complications were obtained by consulting specialists and then calculating the complication cost based on the published prices of drugs and tests at the Cancer Hospital of The University of Chinese Academy of Sciences.

### Utility

Utility was assessed by performing a cross-sectional study from April 6, 2021 to May 6, 2021. We chose the EuroQol five-dimensional (EQ-5D-5L) questionnaire to assess patients with PICCs or PORTs. The EQ-5D-5L scale has the highest rate of citation and recommendation in national guidelines, and the 5L questionnaire is more sensitive and accurate than the 3L questionnaire for measuring health status (17–19). Respondent health utility values were calculated according to the Chinese EQ-5D-5L point system formula (20), with higher scores representing better health-related quality of life.

### Cost-utility analysis

In this study, incremental cost-utility-ratio (ICUR) was calculated to compare the cost-utility of PICC and PORT under the threshold of willingness-to-pay (WTP). If the ICUR was less than the WTP, PORT was considered more cost-effective than PICC. If the ICUR was greater than the WTP, PORT was not more cost-effective than PICC.


$$\begin{array}{c} ICER=\frac{COST_{PORT}-COST_{PICC}}{QALY_{PORT}-QALY_{PICC}} \end{array}$$

### Sensitivity analyses

We performed sensitivity analyses to evaluate the uncertainty and robustness of the base-case result. A one-way sensitivity analysis was used to assess the cost of PICC and PORT insertion, maintenance cost, complication rates and health utility values. The range of PICC and PORT costs was obtained from physician surveys, and complication and utility rates were obtained from prior literature. In the probabilistic sensitivity (PSA) analysis, 1000 Monte Carlo simulations were performed based on the distribution of the parameters. The range and distribution of these parameters are shown in Table 2.

**Table 2:** Distribution type and input values for the sensitivity analysis.

**Groups**	**Variable**	**Base-case value (**¥**)**	**Range in the sensitivity analysis**	**Distribution used in the probabilistic sensitivity analysis**
PICC	Insertion cost	1,986.22	1,377.5~2,169.5	Gamma
	Maintenance cost	1,982.85	1,624.13~2,236.67	Gamma
	Thrombosis cost	2,244.98	1,330.36~3,159.6	Gamma
	Infection cost	2,158.44	1,245.48~4,212	Gamma
	Incidence of catheter-related thrombosis	2.50%	2.5~11%	Beta
	Incidence of catheter occlusion	5.90%	1~8%	Beta
	Incidence of migration	5.90%	1~8%	Beta
	Utility	0.92	0.9~0.94 (95%CI)	Beta
PORT	Insertion cost	3,546.37	2,837.1~4,255.64	Gamma
	Maintenance cost	923.72	547.5~1,108	Gamma
	Thrombosis cost	2,244.98	1,330.36~3,159.6	Gamma
	Infection cost	2,158.44	1,245.48~4,212	Gamma
	Incidence of catheter-related thrombosis	1.50%	1.5~8%	Beta
	Incidence of catheter occlusion	4%	0.5~4.8%	Beta
	The incidence of infection	1.50%	1.5~8%	Beta
	Utility	0.95	0.94~0.96 (95%CI)	Beta

*PICC, peripherally inserted central catheter; PORT, Centrally inserted totally implanted access port*.

## Result

### Patients

To reduce selection bias and balance patient baseline characteristics, participants were matched 1:1 for age, gender and diagnosis using a propensity match score (PSM) with a caliper value of 0.005 (21). A difference was considered statistically significant if *P* < 0.05 (14). A *t*-test, chi-square test or Fisher's exact test was used to compare the baseline characteristics of the patients matched by PSM. A total of 404 patients were included after PSM matching, 202 patients in each group. Patient baseline characteristics are shown in Table 3.

**Table 3:** Demographic characteristics.

		**All patients**			**Patients after PSM**		
		**PICC**	**PORT**	***p*** **-value**	**PICC**	**PORT**	***p*** **-value**
		*n =* 313 (%)	*n =* 273 (%)		*n =* 202 (%)	*n =* 202 (%)	
Age		57.54 ± 11.60	57.23 ± 10.84	0.513^α^	57.61 ± 11.02	57.45 ± 10.74	0.802^α^
Sex	Male	184 (58.79)	90 (32.97)	<0.05^β^	77 (38.12)	77 (38.12)	>0.05^β^
	Female	129 (41.21)	183 (67.03)		125 (61.88)	125 (61.88)	
Diagnosis	GI cancer	56 (17.89)	33 (12.09)	<0.05^β^	38 (18.81)	39 (19.3)	0.91^β^
	Lung cancer	72 (23.00)	36 (13.19)		44 (21.78)	35 (17.33)	
	Gynecological cancer	39 (12.46)	64 (23.44)		38 (18.81)	26 (12.87)	
	Breast cancer	23 (7.35)	98 (35.90)		22 (10.89)	65 (32.18)	
	Nasopharyngeal carcinoma	47 (15.02)	0 (0)		19 (9.41)	0 (0)	
	Other	76 (24.28)	42 (15.38)		41 (20.3)	37 (18.32)	

*PICC, peripherally inserted central catheter; PORT, centrally inserted totally implanted access port; GI, gastrointestinal;**α**, Student t-tests; **β**, X^2^-test; PSM, Propensity score matched*.

### Cost

There was a significant difference in the dwell time of PICCs vs. PORTs [PICC (143.4 ± 7.5), PORT (337.6 ± 5.4), *P* < 0.01], and the maintenance cycle of PICCs and PORTs was different. PICCs were maintained once a week while PORTs could be maintained once a month. The average daily maintenance costs of PICCs and PORTs were therefore calculated at 6 months and 12 months with tubes, respectively.

### Utility

A questionnaire survey was performed on 104 patients with PICCs and 91 patients with PORTs for long-term intravenous drug administration. Utility values were higher in the PORT group (0.95) than in the PICC group (0.93, *p* < 0.05), which was similar to what was reported by a previous study (17). Findings are shown in Table 4.

**Table 4:** Utility of PICC and PORT.

**Group**	**Number**	**Mean**	**SE**	***P*** **-value**
PICC	104	0.92	0.0938	*F* = 18.211
PORT	91	0.95	0.0595	*P* <0.01

*PICC, peripherally inserted central catheter; PORT, Centrally inserted totally implanted access port*.

### Cost-utility analyses

Patients who had a PICC for 6 months had a total cost of ¥4,091.7 and 0.46 QALYs, while patients who had a PORT for 6 months incurred a total cost of ¥4,566.8 and 0.475 QALYs. Patients with a PICC for 12 months had a total cost of ¥6,089.6 and 0.92 QALYs, while patients with a PORT for 12 months had a total cost of ¥5,497.5 and 0.95 QALYs. The cost of using a PICC for 12 months was greater than that of a PORT, making PORT the better option with respect to both cost and utility. The results of the economic analysis of using a PICC and PORT for 6 months are shown in Table 5.

**Table 5:** Base-case result.

**Group**	**Cost (**¥**)**	**Effect (QALYs)**	**Incremental cost (**¥**)**	**Incremental effect (QALY)**	**ICUR (**¥**/QALY)**
PICC	4,091.709473	0.46			
PORT	4,566.772369	0.475	475.0628962	0.015	31,670.85975

*QALY, quality-adjusted life-year; ICUR, incremental cost-utility ratio*.

### Sensitivity analyses

**Figure 1:**
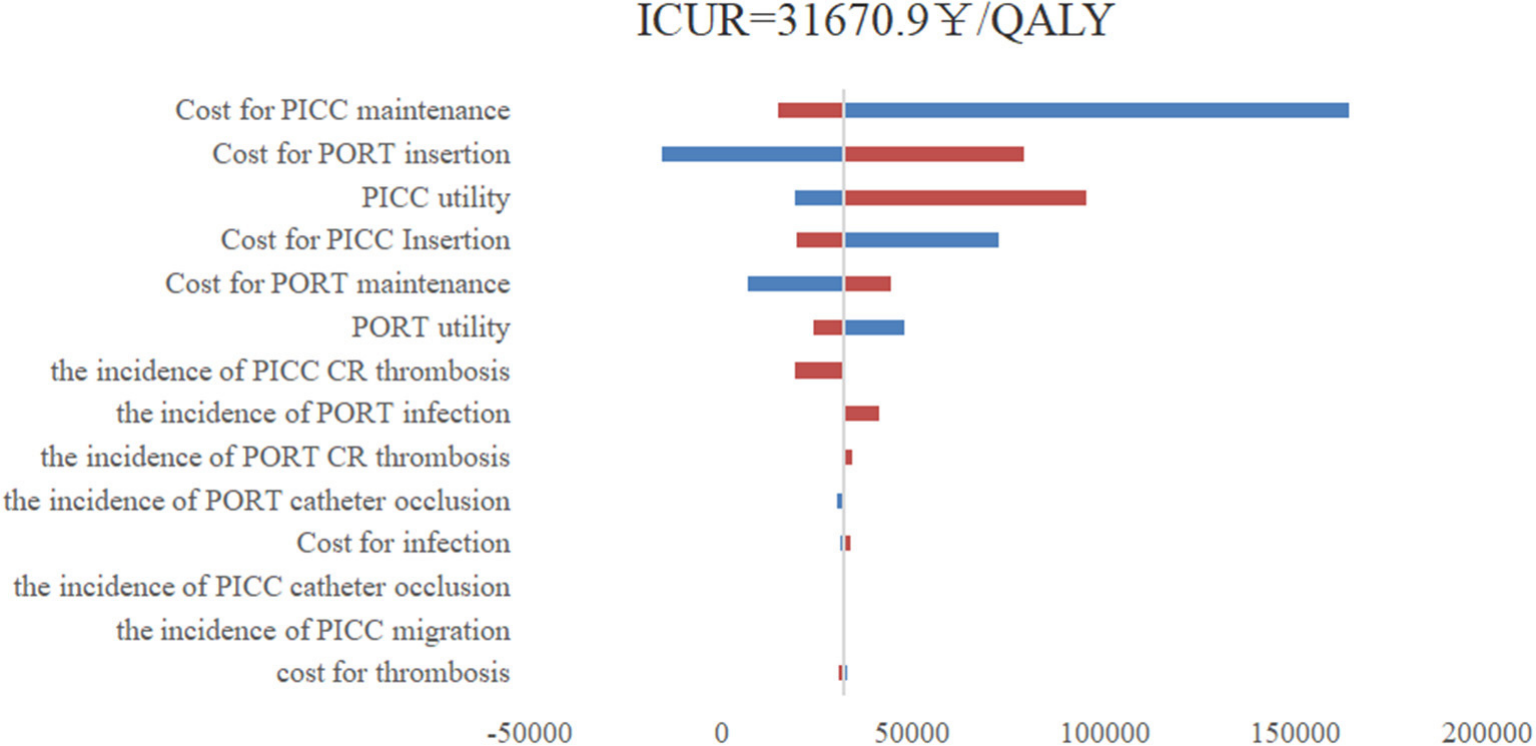
One-way sensitive analysis; PICC, peripherally inserted central catheters; PORT, centrally inserted totally implanted access port; QALY, quality-adjusted life -year; ICUR, incremental costutility ratio; CR, catheter-related.

**Figure 2:**
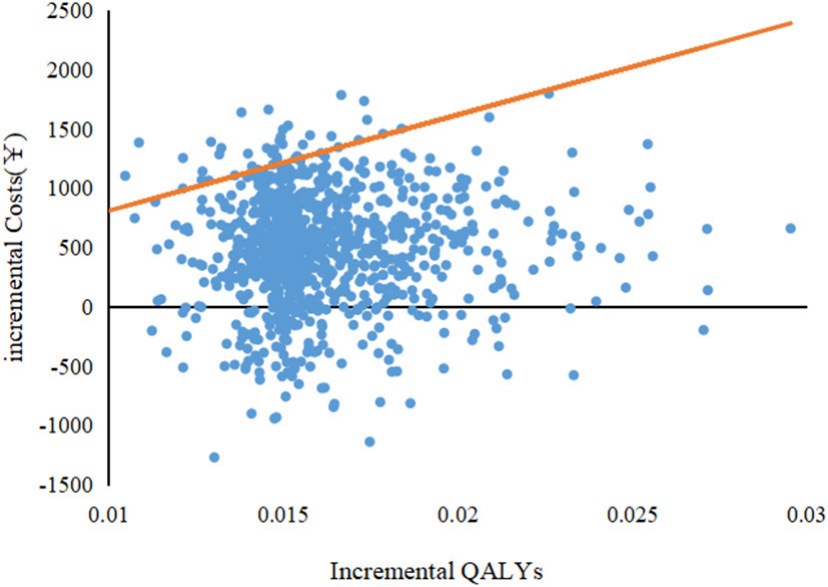
Probabilistic sensitivity analysis; QALY, quality-adjusted life-year.

**Figure 3:**
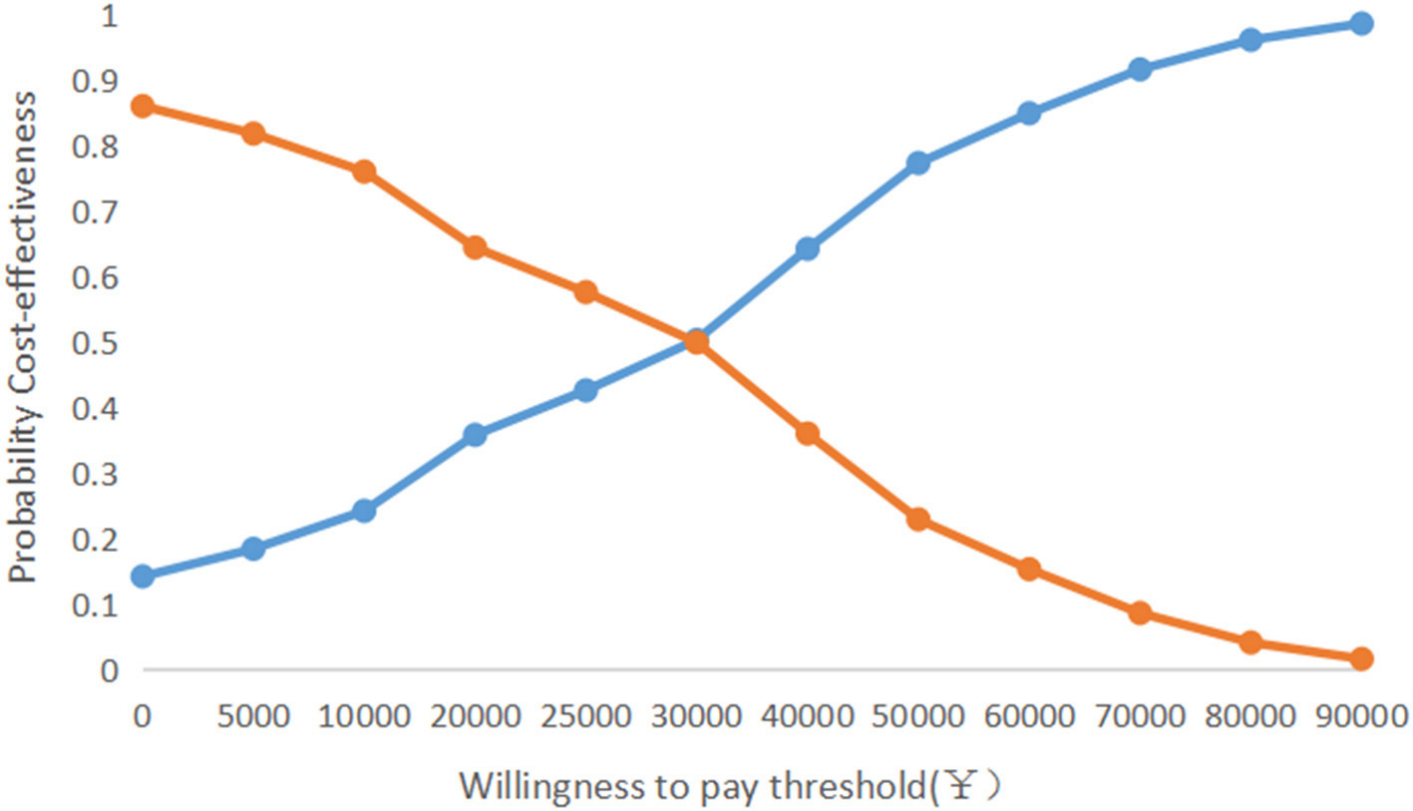
Cost-effectiveness acceptability curve.

As shown in Figure 1, the one-way sensitivity analysis shows that all uncertainties vary within reasonable limits, with the maintenance cost of using a PICC having the greatest impact on the results of the underlying analyses. The PSA results show that under a WTP = 80,976¥/QALY (China's GDP per capita in 2021) threshold, the probability of a PORT being more economical was 96.2%. The cost-effectiveness acceptability curve shows that the probability of a PORT being economical at WTP = 30,000¥/QALY is 50%, and the probability of PORT being cost-effective when WTP was double GDP per capita was 96% (Figures 2, 3).

## Discussion

This study provides a comparative health economics analysis of the costs and health outcomes of PORTs and PICCs as medium and long-term intravenous access for oncology patients from a healthcare system perspective. Although the total insertion cost of a PORT was higher than that of a PICC, due to the high maintenance cost of PICCs and the high incidence of complications, the ICUR of PICCs vs. PORTs was 31,670.9 ¥/QALY at 6 months of intravenous administration. Under the WTP we set (2021 GDP per capita), the use of a PORT was economical. At 12 months of intravenous administration, PORTs were the overwhelmingly superior solution.

In our one-way sensitivity analyses, the maintenance cost of PICCs had the greatest impact on our results, followed by the insertion cost of a PORT, the utility of using a PICC, the insertion cost of a PICC and the maintenance cost of a PORT. The cost of PICCs and PORTs were the main factors that affected their economic results, in particular the maintenance cost of the intravenous infusion device, which accumulated over time. The insertion cost of the PORT was higher than that of the PICC, but the PICC had a shorter maintenance cycle and costs therefore accrued quickly. PORTs therefore became more economical as the duration of use increased.

A cost-utility analysis of PORTs and PICCs was previously performed in China. Wang et al. found that the cost-effectiveness ratio of full PICC placement was lower than that of a PORT when the catheter was left in place for ≤12 months (15), and that the cost-effectiveness ratio was better over this period. The different results of our work may be due to the significant reduction in the cost of PORTs over time and the different evaluation perspectives (provider perspectives) adopted by the two studies. Our study is consistent with the findings of Litian et al. (16), who used a similar evaluation approach to analyze the costs and health outcomes of the full PICC and PORT retention process from a social perspective. However, the data for that study was derived from a meta-analysis and the PORT had not yet experienced a significant price reduction at the time of publication.

This study has the following limitations. First, at the time that this study was conducted, there was a high rate of withdrawal of PICC patients in the short term due to complications or the end of treatment. This may have affected the collection of complications associated with PICCs at a later stage and led to an artificially low reported incidence of PICC complications. Second, utility was collected via a cross-sectional survey with a small sample size. Assessing the utility of patients who are bedridden or have limited mobility makes it unclear if different intravenous delivery devices will have an appreciable impact on their quality of life. A future multicenter health economics study may yield more accurate results.

## Conclusion

This study investigated the economics of two intravenous infusion devices, PICC and PORT, for a 6 and 12-month indwelling time using a cost-utility method based on real-world individual patient data. We found that despite the high cost of a PORT, patients had a higher quality of life and fewer adverse events, making it economical for a 6-month indwelling time. At 12 months the cumulative cost of a PORT was lower than that of a PICC. PORTs also had superior health outputs than PICCs, making it an absolutely superior option. The results of this study provide a theoretical basis for preferentially recommending PORTs as intravenous infusion conduits.

## Data Availability Statement

The original contributions presented in the study are included in the article/supplementary material, further inquiries can be directed to the corresponding author.

## Ethics Statement

The studies involving human participants were reviewed and approved by the Cancer Hospital of the University of Chinese Academy of Sciences (IRB-2020-11). The patients/participants provided their written informed consent to participate in this study.

## Author contributions

The conception and design of this study were primarily conducted by GS. The drafting of the article was mainly the responsibility of XZ. All authors have reviewed the analysis, interpretation of the data, contributed to the drafting of the manuscript, revised the manuscript for important intellectual content, approved the final version to be published, and agree to be accountable for all the aspects of this study.

## Funding

This study was financially supported by Zhejiang Provincial Health Commission (2020KY063).

## Conflict of interest

The authors declare that the research was conducted in the absence of any commercial or financial relationships that could be construed as a potential conflict of interest.

## Publisher's note

All claims expressed in this article are solely those of the authors and do not necessarily represent those of their affiliated organizations, or those of the publisher, the editors and the reviewers. Any product that may be evaluated in this article, or claim that may be made by its manufacturer, is not guaranteed or endorsed by the publisher.
